# Extracorporeal membrane oxygenation (ECMO) for critically ill adults in the emergency department: history, current applications, and future directions

**DOI:** 10.1186/s13054-015-1155-7

**Published:** 2015-12-17

**Authors:** Jarrod M. Mosier, Melissa Kelsey, Yuval Raz, Kyle J. Gunnerson, Robyn Meyer, Cameron D. Hypes, Josh Malo, Sage P. Whitmore, Daniel W. Spaite

**Affiliations:** Department of Emergency Medicine, University of Arizona, 1609 N. Warren Ave, Tucson, AZ 85724 USA; Arizona Emergency Medicine Research Center, University of Arizona, 1609 N. Warren Ave, Tucson, AZ 85724 USA; Division of Pulmonary, Critical Care, Allergy and Sleep, Department of Medicine, University of Arizona, 1501 N Campbell Ave, Tucson, AZ 85721 USA; Division of Pediatric Intensive Care, Department of Pediatrics, University of Arizona, 1501 N Campbell Ave, Tucson, AZ 85721 USA; Division of Emergency Critical Care, Department of Emergency Medicine, University of Michigan Health System, 1500 E. Medical Center Drive, Ann Arbor, MI 48109 USA

## Abstract

Extracorporeal membrane oxygenation (ECMO) is a mode of extracorporeal life support that augments oxygenation, ventilation and/or cardiac output via cannulae connected to a circuit that pumps blood through an oxygenator and back into the patient. ECMO has been used for decades to support cardiopulmonary disease refractory to conventional therapy. While not robust, there are promising data for the use of ECMO in acute hypoxemic respiratory failure, cardiac arrest, and cardiogenic shock and the potential indications for ECMO continue to increase. This review discusses the existing literature on the potential use of ECMO in critically ill patients within the emergency department.

## Introduction

Extracorporeal life support (ECLS) is a general term used to describe temporary support of cardiac or pulmonary function using mechanical devices. When using the “heart–lung machine” to completely bypass the cardiopulmonary circulation, it is referred to as cardiopulmonary bypass. When ECLS is used in the intensive care unit (ICU) or emergency department (ED) to augment oxygenation, ventilation, or cardiac output it is generally referred to as extracorporeal membrane oxygenation (ECMO). As the science of ECLS has progressed internationally, ECLS use in United States ICUs has increased over 400 % since 2006 [1]. This has led to interest in potential earlier applications in the ED, and possibly even the prehospital setting. This review focuses on one modality of ECLS, ECMO, and describes the history of ECMO, common current applications, and important considerations related to ECMO use in adult ED patients.

## History

ECLS was initially developed in the 1950s by John Gibbon as a means of oxygenating blood via a membrane oxygenator during prolonged operations on cardiopulmonary bypass [2]. Given the lack of an open reservoir of blood and extreme anticoagulation required with a traditional cardiopulmonary bypass circuit, ECMO presented a less complex and more sustainable option for treatment of refractory cardiovascular and respiratory failure outside the operating room. Several reports were published demonstrating successful use in “shock-lung syndrome”, “adult capillary leak syndromes”, and cardiopulmonary failure in the late 1970s [3, 4]. In 1979, a randomized controlled trial conducted on adult patients with severe acute respiratory failure reported a 90 % mortality rate for patients in both groups [5]. Thus, enthusiasm stalled and over the next 30 years ECMO was used mostly for neonatal and pediatric patients with only a small number of highly specialized centers pursuing ECMO in adult patients.

Technological improvements and advances in other aspects of critical care have created an ECMO landscape that is very different from the early days of 90 % mortality. For patients with severe hypoxemic respiratory failure, the publication of the Conventional Ventilatory Support versus Extracorporeal Membrane Oxygenation for Severe Adult Respiratory Failure (CESAR) trial and several reports of improved survival in patients with acute respiratory distress syndrome (ARDS) treated with ECMO during the H1N1 influenza pandemic have led to a significant expansion of ECMO use [6–9]. Recent data show promise when ECMO is used early in patients with cardiac arrest to augment traditional cardiopulmonary resuscitation in the form of extracorporeal cardiopulmonary resuscitation (ECPR) [10–15]. ECPR may play a role in the prehospital [16] or ED management of refractory cardiac arrest, cardiovascular collapse due to pulmonary embolism [17], hypothermia [18], drowning [19], overdoses [20], airway obstruction [21], and severe electrolyte abnormalities [22].

## Current applications

### Veno-venous ECMO for severe acute respiratory failure

Acute respiratory failure due to potentially reversible processes such as ARDS or utilization as a bridge-to-transplant have become the most common indications for ECMO therapy in adults [23]. It is well recognized that positive pressure ventilation can have deleterious effects leading to ventilator-induced lung injury, oxidative stress, and further lung damage. Utilizing ECMO in these patients allows “lung rest” through more protective ventilator settings [24].

Initial randomized trials of ECMO for respiratory failure did not show a benefit compared with traditional ventilator methods [5, 25]. In the 1990s and 2000s, survival rates reported in case series with ECMO improved to 52–75 % [26, 27]. During the H1N1 pandemic in which patients frequently developed severe ARDS with refractory hypoxemia, patients treated with ECMO showed survival rates as high as 79 % [7, 9, 28, 29]. The first modern randomized controlled trial of ECMO for adult patients with ARDS, the CESAR trial, was published in 2009. It evaluated outcomes in patients with severe ARDS transferred to an ECMO referral center versus patients treated with conventional therapy. Although mortality at any point was not significantly different, the study identified significantly higher 6-month survival rates in the group transferred to the ECMO referral center—of which only 75 % of the patients received ECMO—versus the control group that were not transferred [30]. Thus, it may have been other aspects of care at the ECMO center, not necessarily the ECMO itself, that led to improved outcomes. The Extracorporeal Membrane Oxygenation for Severe Acute Respiratory Distress Syndrome (EOLIA) trial is currently underway, evaluating early ECMO within 3 hours of initiation of mechanical ventilation for patients with refractory hypoxemia and severe ARDS [31]. If this trial finds a benefit of early ECMO for ARDS, coordinating transfer to ECMO-capable referral centers directly from the ED for early initiation of ECMO support may become important for patient outcomes [32]. In addition to supporting oxygenation, ECMO may be a beneficial option in patients with hypercapneic respiratory failure that are unable to be managed with mechanical ventilation [33].

### Veno-arterial ECPR for cardiac arrest

Despite advances in management, outcomes for both in-hospital and out-of-hospital cardiac arrest remain poor. In-hospital cardiac arrest treated with conventional cardiopulmonary resuscitation (CPR) typically has a survival rate of 15–17 % and out-of-hospital cardiac arrest (OHCA) survival is even lower at only 8–10 % [34, 35]. The worst outcomes are in patients with prolonged time to return of spontaneous circulation. Prolonged cerebral hypoperfusion leads to significantly worse neurologic sequelae and early initiation of ECPR with veno-arterial (VA) ECMO may be a useful adjunct to reducing the interval time from arrest to restoration of cerebral perfusion.

Data for in-hospital arrest are the most promising, likely due to the shorter interval from the onset of arrest to initiation of ECMO flow. While there are no randomized trials to date, observational studies have reported an association between ECPR and improved survival. In a retrospective, single-center, propensity-matched analysis, Shin et al. [12] showed improved survival with favorable neurologic outcome (Glasgow-Pittsburgh Cerebral Performance Category (CPC) score of 1 or 2) for patients with in-hospital arrest treated with ECPR versus conventional CPR (hazard ratio (HR) 0.17, 95 % confidence interval (CI) 0.04–0.68). Chen et al. [11] found similar 30-day (HR 0.47, 95 % CI 0.28–0.77) and 1-year (HR 0.53, 95 % CI 0.33–0.83) survival for ECPR when compared with conventional CPR. Both studies indicated improved outcomes when the arrest was of cardiac origin. Another retrospective review of prolonged in-hospital arrests (>15 minutes) or refractory shock after return of spontaneous circulation showed nearly half of the patients who survived had a CPC score of 1 or 2 with the use of ECPR [10]. Other observational studies have found variable improvements in mortality with the use of ECPR [36, 37]. A recent meta-analysis performed by Cardarelli et al. [38] in 135 patients from 1990 to 2007 showed a hospital survival rate to discharge with ECPR of 40 %.

Reports from OHCA studies are not as robust, although there are dramatic reports of otherwise hopeless cases rescued by ECMO [39]. Haneya et al. [40] compared ECPR initiated in the ED for OHCA with ECPR initiated for in-hospital cardiac arrest, and found a survival rate of 42 % for in-hospital arrest patients versus only 15 % for OHCA patients. ECPR combined with therapeutic hypothermia and intra-aortic balloon pump placement was recently shown to improve neurologic outcomes for OHCA patients with ventricular fibrillation or pulseless ventricular tachycardia. This study demonstrated survival with a favorable CPC score in 11.2 % of those with ECPR versus 2.6 % with conventional CPR at 6 months [14]. Similar results were reported in a small observational pilot study in Australia combining mechanical compression devices, ECPR, and hypothermia for patients with refractory cardiac arrest. In this small study, 5 of 11 OHCA patients and 9 of 15 in-hospital cardiac arrest patients survived, and favorable neurological outcome was achieved in half of the survivors [15].

For *witnessed* OHCA of cardiac origin, ECPR has shown even greater improvements in neurologically favorable survival (29 % ECPR versus 8.9 % CPR) [13]. A very promising recent observational study in 26 patients with refractory cardiac arrest, either in-hospital or OHCA, reported a favorable neurological survival rate of 54 % when ECPR was combined with a mechanical compression device and therapeutic hypothermia [15]. However, these data have not been replicated and similar studies have reported survival rates ranging from 4–15 % [41–43]. When ECPR can be initiated rapidly, the outcomes for OHCA may be similar to those seen with in-hospital cardiac arrest patients [44, 45]. Although there are no large prospective randomized trials and survival from refractory cardiac arrest is poor, ECPR may provide a tool to improve survival with good neurologic outcomes when initiated early in selected patients.

While an optimistic estimate of survival from OHCA with the use of ECPR may be in the 15–20 % range, the critical factor that determines success appears to be the duration from the onset of arrest to achieving ECMO flow. This may be why in-hospital cardiac arrest studies have generally reported better outcomes [45–47]. Furthermore, the volume of patients meeting optimal criteria for ECPR, including witnessed ventricular fibrillation/tachycardia with a short interval to initiation of CPR, refractory arrest despite optimal resuscitative efforts, and <75 years of age comprises a very small (<10 %) subset of all patients with OHCA [48, 49]. Additionally, complication rates of ECPR remain high [43, 50, 51]. Currently the American Heart Association’s position is that evidence does not support a recommendation for ECPR, although it may be considered in highly specialized centers in patients who have a potentially reversible disease and a short duration of cardiac arrest [52]. The recent Institute of Medicine report on cardiac arrest care states ECMO is an emerging technology that has promise in improving cardiac arrest care and should be developed and researched [53].

### VA ECMO for shock

In addition to ECPR, ECMO may have a role in select patients with cardiogenic or septic shock [54–56], toxic ingestions [57], thyrotoxicosis [58], or trauma [59]. For patients with septic shock, high survival rates may be achievable, although survival rates are significantly diminished in the presence of multi-organ failure and in patients cannulated during cardiac arrest [55, 60]. ECMO has shown promising results as a method of supporting hemodynamics in patients with cardiogenic shock due to acute myocardial infarction [61], massive pulmonary embolism [62] or myocarditis [63]. ECMO may be preferable to aortic balloon pumps as it can provide more robust biventricular support by increasing right ventricular drainage in addition to supporting gas exchange, and it can be employed quickly at the bedside. However, the downside of ECMO for cardiac support is that because of the retrograde aortic flow, left ventricular afterload and oxygen demand increase without the placement of a left ventricular drain. ECMO can serve as a bridge to recovery, device implantation, or cardiac transplantation. Small trials have shown improved survival rates in patients placed on ECMO as a bridge to a left ventricular assist device and subsequently to transplantation [64]. Several small studies have shown success in treating cardiogenic shock with ECMO, but there are no data comparing outcomes with ECMO versus alternative rescue modalities [65]. Arranging the initiation of ECMO may be a viable option in patients with known severe cardiac dysfunction in shock refractory to conventional therapy in the ED.

## Important considerations

### Programmatic considerations

A successful ECLS program requires a significant multidisciplinary and organizational commitment to ensure necessary resources and personnel [66]. Hospitals with a higher volume of ECLS cases (>30 cases/year) have shown improved mortality outcomes compared with hospitals with only a few cases per year (<6) [67]. Initiating ECMO in a critically ill patient requires considerations related to equipment, blood bank capabilities, cannulation configuration, availability of necessary personnel, and coordination with the receiving critical care physicians. In a recent trial evaluating ECMO for OHCA, initiation of ECMO required two critical care physicians for cannulation, a third physician providing ultrasound guidance of cannula placement in the inferior vena cava, a fourth physician dedicated to leading the resuscitation, a nurse coordinator to initiate the circuit, and a sixth person to infuse cold saline for intra-arrest hypothermia [15]. Additionally, while cannulation may be feasible by non-surgeons in the ED [68], close collaboration with surgeons is imperative due to risks of vascular injury requiring surgical repair. A successful ECLS program requires physicians, nurses, perfusionists, and respiratory therapists trained and competent in cannulation and management of the ECLS circuit to be available in sufficient numbers to provide 24/7 coverage. A streamlined exit strategy from the ED should be established, whether that includes transfer to the cardiac catheterization lab, ICU, or transfer to an ECMO receiving hospital. Predetermined inclusion and exclusion criteria, as well as guidelines on how and when to separate from ECMO in a patient who fails to improve are important to optimize outcomes and resource utilization. Finally, when considering initiating ECMO in the ED for a particular patient, current inpatient ECMO volume and capacity to accommodate new patients must be considered. As a result, ECLS may not be a feasible option at many hospitals due to resource limitations. Local, regional, and inter-regional networks of hospitals with direct communication with the ECMO referral center that provides a mobile rescue team for cannulation and transport of patients with critical cardiopulmonary failure may be the optimal solution to alleviate many of these costly programmatic considerations.

### Technical considerations

**Fig. 1:**
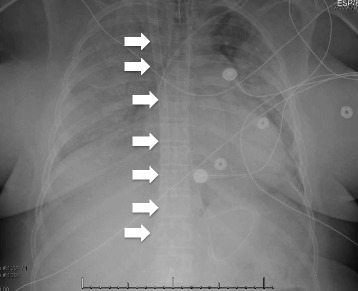
Veno-venous cannulation for ARDS. This chest X-ray demonstrates severe airspace disease in a patient with ARDS. The dual-lumen ECMO cannula (*arrows*) can be seen passing through the internal jugular vein, superior vena cava, and terminating in the inferior vena cava at the level of the hepatic vein

**Fig. 2:**
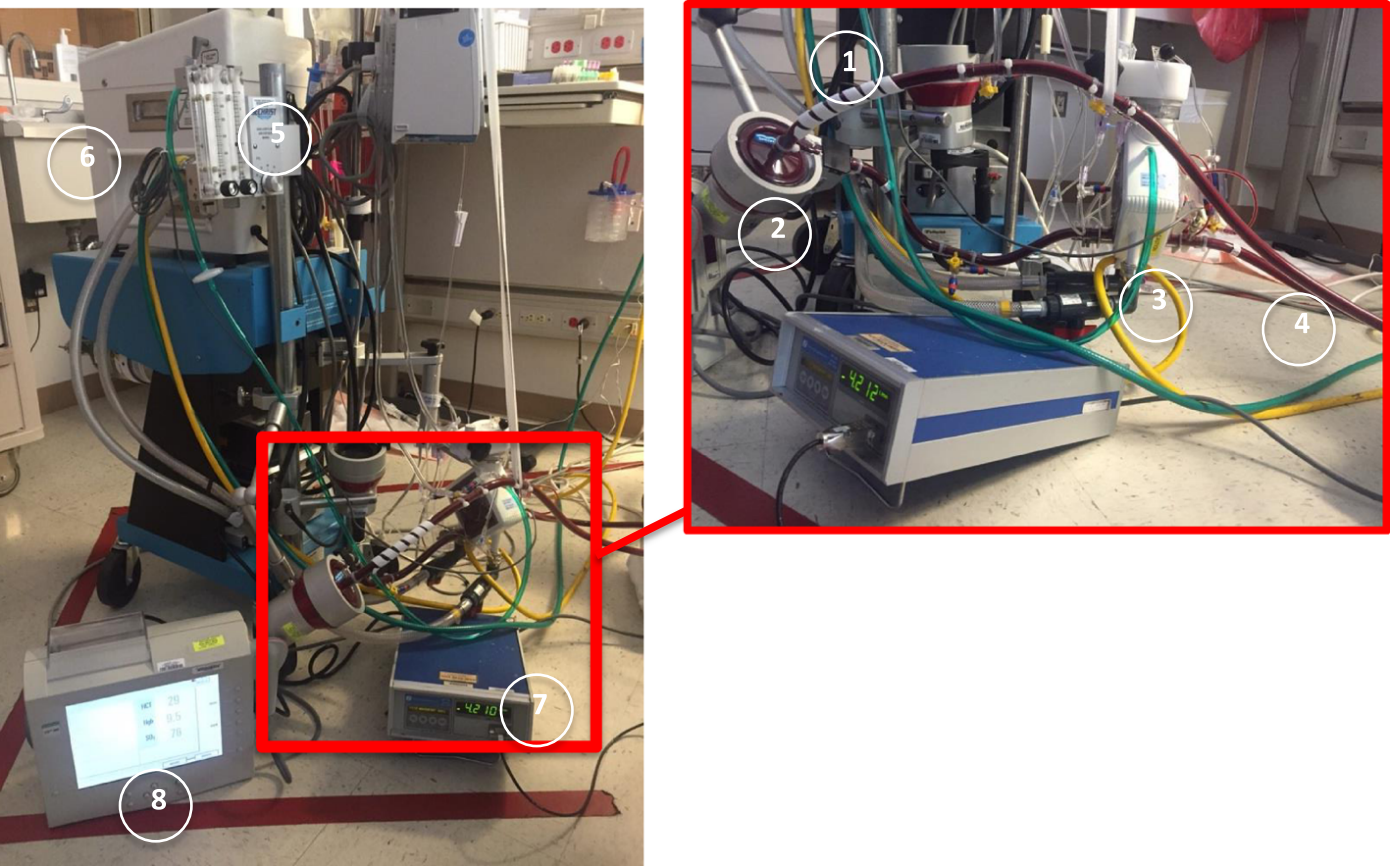
ECMO circuit components and flow. Flow through the ECMO circuit starts with the venous drainage cannula (1), which is propelled forward by the pressure gradient generated by the centrifugal pump head (2). The blood passes through the oxygenator (3) and then returns to the patient in the outflow tubing back into the right atrium (VV) or femoral artery (VA) (4). Gas exchange is regulated by the amount of countercurrent “sweep” gas flowing through the oxygenator (5) and the blood is warmed by the thermoregulator attached to the circuit (6). Flow, hemoglobin, hematocrit and venous saturation can be continuously monitored by ultrasonic meters attached to the circuit (7 and 8)

Cannulation strategies must be carefully considered based on the underlying pathophysiology. Configurations include veno-venous (VV) and VA approaches, with hybrid modes possible for specialized uses (e.g., veno-arterial-venous for patients with cardiogenic shock and differential hypoxemia). In VV ECMO, patients are typically cannulated through the femoral vein and internal jugular/superior vena cava with two cannulas, or a single dual-lumen cannula placed into the right atrium and inferior vena cava via the right internal jugular vein (Fig. 1). Venous blood is drained from the vena cava regardless of the cannulation configuration. After passing through the oxygenator, blood is returned to the right atrium via the return cannula (Fig. 2). This configuration requires blood to pass through the pulmonary circulation and is systemically circulated by the native cardiac output. Thus, VV ECMO provides no cardiac support.

Percutaneous VA ECMO in adults most commonly involves femoral artery and vein cannulation where oxygenated blood is returned to the femoral artery and flows retrograde into the aorta. Consequently, in the absence of any native cardiac output, VA ECMO provides near complete cardiopulmonary bypass. In the presence of native cardiac output, blood flow out of the left ventricle mixes with retrograde ECMO return flow in the aorta. If there is no intrapulmonary shunt, the native cardiac output is well oxygenated and this mixing is not detrimental. However, if the patient’s lungs are failing (e.g., because of ARDS), blood flow from the native cardiac output is deoxygenated and competes with retrograde flowing oxygenated blood from the ECMO circuit. These competing flows mix somewhere in the aorta and can create differential hypoxemia (“North–south” syndrome) whereby poorly oxygenated blood preferentially perfuses the upper body, including the brain and heart, potentially leading to coronary and cerebral ischemia.

## Contraindications, complications, and ethical challenges

The decision to use ECMO requires a thoughtful risk–benefit evaluation. Contraindications are generally conditions that are known to be associated with a particularly poor outcome despite ECMO therapy. Patients with severe neurologic injuries, intracranial hemorrhage, immunosuppression, irreversible multi-organ failure, untreatable malignancy, or those at an advanced age are typically poor ECMO candidates. Patients with aortic dissections or severe aortic regurgitation are not ECMO candidates due to the risks of propagating the dissection and over-distending the left ventricle, respectively. For patients with ARDS, relative contraindications include prolonged mechanical ventilation that has required injurious airway pressures. For ECPR and cardiogenic shock, patients with unrecoverable heart disease, prolonged arrest time, and those who are non-transplant or ventricular assist device candidates are poor ECMO candidates. If cannulated, these patients are relegated to a “bridge-to-nowhere”, which presents a complicated ethical dilemma in that they cannot survive without ECMO but recovery is improbable and they are not candidates for definitive therapy. Patients generally require anticoagulation while on ECMO and, while it is not an absolute contraindication, an inability to anticoagulate complicates both cannulation and long-term management strategies.

Complication rates with ECMO are high. This is true during both cannulation and ongoing management [69]. Complications include hemorrhage, stroke, limb ischemia, thrombosis, and infection from the indwelling lines/tubes. Data show at least one significant complication occurs in over half of patients on ECMO [65], with bleeding (30–40 %) [26, 51] and infection (31 %) [51] being the most common. Hemorrhagic complications most commonly occur at the cannulation or surgical sites themselves and are generally related to anti-coagulation, of which VA ECMO requires more aggressive anticoagulation due to the risk of arterial thrombosis. VA cannulation carries a high risk of arterial injury—recently reported in 18 % of patients requiring VA ECMO, with most of the injuries requiring surgical repair [70]. Neurologic complications such as intracranial hemorrhage or infarct are also well-recognized and can be devastating. Other complications include hemolysis, pulmonary edema, and lower extremity ischemia from occlusion of the arterial flow with placement of the arterial cannula, which can be prevented with the routine placement of an antegrade arterial cannula to that limb.

Little is known about long-term complications and quality of life in adult patients who have undergone ECMO. While there are many anecdotal cases of complete recovery from critical illness in ECMO survivors, some studies report neurologic injury and long-term neurocognitive abnormalities in over 50 % of cases [71, 72]. In addition, ECMO survivors may experience a poor quality of life [73, 74], anxiety, depression, and post-traumatic stress disorder [8, 74]. ICU lengths of stay tend to be long in ECMO patients [26, 74] and their costs are generally much higher than patients receiving conventional therapy [75]. The challenges of complications, cost, and resource utilization, when taken together with the potential of creating a bridge-to-nowhere situation for some patients and lack of high-quality evidence, create an ethical obligation to consider the risks and benefits very carefully when considering ECMO in a patient’s treatment plan.

## Conclusion

Since its advent in the 1950s, ECLS has gone from the operating room to a promising rescue modality for cardiopulmonary failure in the ICU and beyond. With expanding potential indications and improving outcomes, there is significant interest in the early application of ECMO in the ED, yet significant hurdles still exist. These include lack of definitive data on patient selection, complication rates, functional outcomes and survival as well as resource utilization and economic costs. The H1N1 pandemic, combined with improvements in general critical care, brought modern and improved ECMO technology into the limelight with good outcomes in the treatment of severe acute respiratory failure. Combined with recent data in patients with cardiac arrest and shock that show promising trends, there appears to be potential for the early application of ECLS to patients in the ED. Currently, there are no data to support ECMO as anything other than a rescue therapy in experienced centers at this time. Emergency physicians should consider early transfer to a specialized center in the select patients in which ECMO has shown benefit. Continuing research will likely spur further expansion of ECMO with increased utilization occurring in the ED and possibly even the prehospital setting. However, we encourage a cautious and evidence-based approach to future applications of ECMO prior to widespread adoption given the logistical and ethical challenges of this technology that has the potential to outpace the supporting data.

## Abbreviations

ARDS: Acute respiratory distress syndrome
CESAR: Conventional Ventilatory Support versus Extracoporeal Membrane Oxygenation for Severe Adult Respiratory Failure
CI: Confidence interval
CPC: Cerebral Performance Category
CPR: Cardiopulmonary resuscitation
ECLS: Extracorporeal life support
ECMO: Extracorporeal membrane oxygenation
ECPR: Extracorporeal cardiopulmonary resuscitation
ED: Emergency department
HR: Hazard ratio
ICU: Intensive care unit
OHCA: Out-of-hospital cardiac arrest
VA: Veno-arterial
VV: Venovenous

## References

[1] Sauer CM, Yuh DD, Bonde P (2015). Extracorporeal membrane oxygenation use has increased by 433 % in adults in the United States from 2006 to 2011. ASAIO J.

[2] Gibbon JH (1954). Application of a mechanical heart and lung apparatus to cardiac surgery. Minn Med.

[3] Hill JD, O'Brien TG, Murray JJ, Dontigny L, Bramson ML, Osborn JJ (1972). Prolonged extracorporeal oxygenation for acute post-traumatic respiratory failure (shock-lung syndrome). Use of the Bramson membrane lung. N Engl J Med.

[4] Bartlett RH, Gazzaniga AB, Fong SW, Jefferies MR, Roohk HV, Haiduc N (1977). Extracorporeal membrane oxygenator support for cardiopulmonary failure. Experience in 28 cases. J Thorac Cardiovasc Surg.

[5] Zapol WM, Snider MT, Hill JD, Fallat RJ, Bartlett RH, Edmunds LH (1979). Extracorporeal membrane oxygenation in severe acute respiratory failure. A randomized prospective study. JAMA.

[6] Schmidt M, Hodgson C, Combes A (2015). Extracorporeal gas exchange for acute respiratory failure in adult patients: a systematic review. Crit Care.

[7] Davies A, Jones D, Bailey M, Beca J, Bellomo R, Australia, New Zealand Extracorporeal Membrane Oxygenation Influenza I (2009). Extracorporeal membrane oxygenation for 2009 influenza A(H1N1) acute respiratory distress syndrome. JAMA.

[8] Luyt CE, Combes A, Becquemin MH, Beigelman-Aubry C, Hatem S, Brun AL (2012). Long-term outcomes of pandemic 2009 influenza A(H1N1)-associated severe ARDS. Chest.

[9] Zangrillo A, Biondi-Zoccai G, Landoni G, Frati G, Patroniti N, Pesenti A (2013). Extracorporeal membrane oxygenation (ECMO) in patients with H1N1 influenza infection: a systematic review and meta-analysis including 8 studies and 266 patients receiving ECMO. Crit Care.

[10] Bednarczyk JM, White CW, Ducas RA, Golian M, Nepomuceno R, Hiebert B (2014). Resuscitative extracorporeal membrane oxygenation for in hospital cardiac arrest: a Canadian observational experience. Resuscitation.

[11] Chen YS, Lin JW, Yu HY, Ko WJ, Jerng JS, Chang WT (2008). Cardiopulmonary resuscitation with assisted extracorporeal life-support versus conventional cardiopulmonary resuscitation in adults with in-hospital cardiac arrest: an observational study and propensity analysis. Lancet.

[12] Shin TG, Choi JH, Jo IJ, Sim MS, Song HG, Jeong YK (2011). Extracorporeal cardiopulmonary resuscitation in patients with inhospital cardiac arrest: a comparison with conventional cardiopulmonary resuscitation. Crit Care Med.

[13] Maekawa K, Tanno K, Hase M, Mori K, Asai Y (2013). Extracorporeal cardiopulmonary resuscitation for patients with out-of-hospital cardiac arrest of cardiac origin: a propensity-matched study and predictor analysis. Crit Care Med.

[14] Sakamoto T, Morimura N, Nagao K, Asai Y, Yokota H, Nara S (2014). Extracorporeal cardiopulmonary resuscitation versus conventional cardiopulmonary resuscitation in adults with out-of-hospital cardiac arrest: a prospective observational study. Resuscitation.

[15] Stub D, Bernard S, Pellegrino V, Smith K, Walker T, Sheldrake J (2015). Refractory cardiac arrest treated with mechanical CPR, hypothermia, ECMO and early reperfusion (the CHEER trial). Resuscitation.

[16] Lamhaut L, Jouffroy R, Soldan M, Phillipe P, Deluze T, Jaffry M (2013). Safety and feasibility of prehospital extra corporeal life support implementation by non-surgeons for out-of-hospital refractory cardiac arrest. Resuscitation.

[17] Yusuff HO, Zochios V, Vuylsteke A (2015). Extracorporeal membrane oxygenation in acute massive pulmonary embolism: a systematic review. Perfusion.

[18] Jarosz A, Darocha T, Kosinski S, Zietkiewicz M, Drwila R (2015). Extracorporeal membrane oxygenation in severe accidental hypothermia. Intensive Care Med.

[19] Kim KI, Lee WY, Kim HS, Jeong JH, Ko HH (2014). Extracorporeal membrane oxygenation in near-drowning patients with cardiac or pulmonary failure. Scand J Trauma Resusc Emerg Med.

[20] St-Onge M, Fan E, Megarbane B, Hancock-Howard R, Coyte PC (2015). Venoarterial extracorporeal membrane oxygenation for patients in shock or cardiac arrest secondary to cardiotoxicant poisoning: a cost-effectiveness analysis. J Crit Care.

[21] Ko M, dos Santos PR, Machuca TN, Marseu K, Waddell TK, Keshavjee S (2015). Use of single-cannula venous-venous extracorporeal life support in the management of life-threatening airway obstruction. Ann Thorac Surg.

[22] Palatinus JA, Lieber SB, Joyce KE, Richards JB (2015). Extracorporeal membrane oxygenation support for hypokalemia-induced cardiac arrest: a case report and review of the literature. J Emerg Med.

[23] Ventetuolo CE, Muratore CS (2014). Extracorporeal life support in critically ill adults. Am J Respir Crit Care Med.

[24] Schmidt M, Stewart C, Bailey M, Nieszkowska A, Kelly J, Murphy L (2015). Mechanical ventilation management during extracorporeal membrane oxygenation for acute respiratory distress syndrome: a retrospective international multicenter study. Crit Care Med.

[25] Morris AH, Wallace CJ, Menlove RL, Clemmer TP, Orme JF, Weaver LK (1994). Randomized clinical trial of pressure-controlled inverse ratio ventilation and extracorporeal CO2 removal for adult respiratory distress syndrome. Am J Respir Crit Care Med.

[26] Brogan TV, Thiagarajan RR, Rycus PT, Bartlett RH, Bratton SL (2009). Extracorporeal membrane oxygenation in adults with severe respiratory failure: a multi-center database. Intensive Care Med.

[27] Lewandowski K, Rossaint R, Pappert D, Gerlach H, Slama KJ, Weidemann H (1997). High survival rate in 122 ARDS patients managed according to a clinical algorithm including extracorporeal membrane oxygenation. Intensive Care Med.

[28] Noah MA, Peek GJ, Finney SJ, Griffiths MJ, Harrison DA, Grieve R (2011). Referral to an extracorporeal membrane oxygenation center and mortality among patients with severe 2009 influenza A(H1N1). JAMA.

[29] Kutlesa M, Novokmet A, Josipovic Mraovic R, Filar B, Mardesic P, Barsic B (2014). Extracorporeal membrane oxygenation treatment for H1N1-induced acute respiratory distress syndrome (ARDS): results of the Croatian Referral Center for Respiratory ECMO. Int J Artificial Organs.

[30] Peek GJ, Mugford M, Tiruvoipati R, Wilson A, Allen E, Thalanany MM (2009). Efficacy and economic assessment of conventional ventilatory support versus extracorporeal membrane oxygenation for severe adult respiratory failure (CESAR): a multicentre randomised controlled trial. Lancet.

[31] Combes A (2011). Extracorporeal membrane oxygenation (ECMO) for severe acute respiratory distress syndrome (ARDS). The EOLIA (ECMO to rescue Lung Injury in severe ARDS) trial: a multicenter, international, randomized, controlled open trial [in French]. Reanimation.

[32] Chiu LC, Tsai FC, Hu HC, Chang CH, Hung CY, Lee CS (2015). Survival predictors in acute respiratory distress syndrome with extracorporeal membrane oxygenation. Ann Thorac Surg.

[33] Kluge S, Braune SA, Engel M, Nierhaus A, Frings D, Ebelt H (2012). Avoiding invasive mechanical ventilation by extracorporeal carbon dioxide removal in patients failing noninvasive ventilation. Intensive Care Med.

[34] Peberdy MA, Kaye W, Ornato JP, Larkin GL, Nadkarni V, Mancini ME (2003). Cardiopulmonary resuscitation of adults in the hospital: a report of 14720 cardiac arrests from the National Registry of Cardiopulmonary Resuscitation. Resuscitation.

[35] McNally B, Robb R, Mehta M, Vellano K, Valderrama AL, Yoon PW (2011). Out-of-hospital cardiac arrest surveillance—Cardiac Arrest Registry to Enhance Survival (CARES), United States, October 1, 2005--December 31, 2010. MMWR Surveill Summ.

[36] Chou TH, Fang CC, Yen ZS, Lee CC, Chen YS, Ko WJ (2014). An observational study of extracorporeal CPR for in-hospital cardiac arrest secondary to myocardial infarction. Emerg Med J.

[37] Siao FY, Chiu CC, Chiu CW, Chen YC, Chen YL, Hsieh YK (2015). Managing cardiac arrest with refractory ventricular fibrillation in the emergency department: conventional cardiopulmonary resuscitation versus extracorporeal cardiopulmonary resuscitation. Resuscitation.

[38] Cardarelli MG, Young AJ, Griffith B (2009). Use of extracorporeal membrane oxygenation for adults in cardiac arrest (E-CPR): a meta-analysis of observational studies. ASAIO J.

[39] Nusbaum DM, Bassett ST, Gregoric ID, Kar B (2014). A case of survival after cardiac arrest and 3(1/2) hours of resuscitation. Texas Heart Institute J.

[40] Haneya A, Philipp A, Diez C, Schopka S, Bein T, Zimmermann M (2012). A 5-year experience with cardiopulmonary resuscitation using extracorporeal life support in non-postcardiotomy patients with cardiac arrest. Resuscitation.

[41] Le Guen M, Nicolas-Robin A, Carreira S, Raux M, Leprince P, Riou B (2011). Extracorporeal life support following out-of-hospital refractory cardiac arrest. Crit Care.

[42] Avalli L, Maggioni E, Formica F, Redaelli G, Migliari M, Scanziani M (2012). Favourable survival of in-hospital compared to out-of-hospital refractory cardiac arrest patients treated with extracorporeal membrane oxygenation: an Italian tertiary care centre experience. Resuscitation.

[43] Johnson NJ, Acker M, Hsu CH, Desai N, Vallabhajosyula P, Lazar S (2014). Extracorporeal life support as rescue strategy for out-of-hospital and emergency department cardiac arrest. Resuscitation.

[44] Wang CH, Chou NK, Becker LB, Lin JW, Yu HY, Chi NH (2014). Improved outcome of extracorporeal cardiopulmonary resuscitation for out-of-hospital cardiac arrest—a comparison with that for extracorporeal rescue for in-hospital cardiac arrest. Resuscitation.

[45] Leick J, Liebetrau C, Szardien S, Fischer-Rasokat U, Willmer M, van Linden A (2013). Door-to-implantation time of extracorporeal life support systems predicts mortality in patients with out-of-hospital cardiac arrest. Clin Res Cardiol.

[46] Park SB, Yang JH, Park TK, Cho YH, Sung K, Chung CR (2014). Developing a risk prediction model for survival to discharge in cardiac arrest patients who undergo extracorporeal membrane oxygenation. Int J Cardiol.

[47] Ryu JA, Cho YH, Sung K, Choi SH, Yang JH, Choi JH (2015). Predictors of neurological outcomes after successful extracorporeal cardiopulmonary resuscitation. BMC Anesthesiol.

[48] Poppe M, Weiser C, Holzer M, Sulzgruber P, Datler P, Keferbock M (2015). The incidence of “load&go” out-of-hospital cardiac arrest candidates for emergency department utilization of emergency extracorporeal life support: a one-year review. Resuscitation.

[49] Patel JK, Schoenfeld E, Parnia S, Singer AJ, Edelman N. Venoarterial extracorporeal membrane oxygenation in adults with cardiac arrest. J Intensive Care Med. 2015. 10.1177/0885066615583651 25922385

[50] Xie A, Phan K, Yi-Chin Tsai M, Yan TD, Forrest P (2015). Venoarterial extracorporeal membrane oxygenation for cardiogenic shock and cardiac arrest: a meta-analysis. J Cardiothorac Vasc Anesth.

[51] Cheng R, Hachamovitch R, Kittleson M, Patel J, Arabia F, Moriguchi J (2014). Complications of extracorporeal membrane oxygenation for treatment of cardiogenic shock and cardiac arrest: a meta-analysis of 1,866 adult patients. Ann Thorac Surg.

[52] Cave DM, Gazmuri RJ, Otto CW, Nadkarni VM, Cheng A, Brooks SC (2010). Part 7: CPR techniques and devices: 2010 American Heart Association Guidelines for Cardiopulmonary Resuscitation and Emergency Cardiovascular Care. Circulation.

[53] Graham R, McCoy MA, Schultz AM. Strategies to improve cardiac arrest survival: a time to act. The National Academies Press. 2015. http://www.nap.edu/catalog/21723/strategies-to-improve-cardiac-arrest-survival-a-time-to-act. 26225413

[54] Kar B, Basra SS, Shah NR, Loyalka P (2012). Percutaneous circulatory support in cardiogenic shock: interventional bridge to recovery. Circulation.

[55] Brechot N, Luyt CE, Schmidt M, Leprince P, Trouillet JL, Leger P (2013). Venoarterial extracorporeal membrane oxygenation support for refractory cardiovascular dysfunction during severe bacterial septic shock. Crit Care Med.

[56] Park TK, Yang JH, Jeon K, Choi SH, Choi JH, Gwon HC (2015). Extracorporeal membrane oxygenation for refractory septic shock in adults. Eur J Cardiothorac Surg.

[57] Weinberg RL, Bouchard NC, Abrams DC, Bacchetta M, Dzierba AL, Burkart KM (2014). Venoarterial extracorporeal membrane oxygenation for the management of massive amlodipine overdose. Perfusion.

[58] Hsu LM, Ko WJ, Wang CH (2011). Extracorporeal membrane oxygenation rescues thyrotoxicosis-related circulatory collapse. Thyroid.

[59] Ried M, Bein T, Philipp A, Muller T, Graf B, Schmid C (2013). Extracorporeal lung support in trauma patients with severe chest injury and acute lung failure: a 10-year institutional experience. Crit Care.

[60] Huang CT, Tsai YJ, Tsai PR, Ko WJ (2013). Extracorporeal membrane oxygenation resuscitation in adult patients with refractory septic shock. J Thorac Cardiovasc Surg.

[61] Esper SA, Bermudez C, Dueweke EJ, Kormos R, Subramaniam K, Mulukutla S (2015). Extracorporeal membrane oxygenation support in acute coronary syndromes complicated by cardiogenic shock. Catheter Cardiovasc Interv.

[62] Northey LC, Shiraev T, Omari A (2015). Salvage intraosseous thrombolysis and extracorporeal membrane oxygenation for massive pulmonary embolism. J Emerg Trauma Shock.

[63] Diddle JW, Almodovar MC, Rajagopal SK, Rycus PT, Thiagarajan RR (2015). Extracorporeal membrane oxygenation for the support of adults with acute myocarditis. Crit Care Med.

[64] Pagani FD, Aaronson KD, Swaniker F, Bartlett RH (2001). The use of extracorporeal life support in adult patients with primary cardiac failure as a bridge to implantable left ventricular assist device. Ann Thorac Surg.

[65] Combes A, Leprince P, Luyt CE, Bonnet N, Trouillet JL, Leger P (2008). Outcomes and long-term quality-of-life of patients supported by extracorporeal membrane oxygenation for refractory cardiogenic shock. Crit Care Med.

[66] Combes A, Brodie D, Bartlett R, Brochard L, Brower R, Conrad S (2014). Position paper for the organization of extracorporeal membrane oxygenation programs for acute respiratory failure in adult patients. Am J Respir Crit Care Med.

[67] Barbaro RP, Odetola FO, Kidwell KM, Paden ML, Bartlett RH, Davis MM (2015). Association of hospital-level volume of extracorporeal membrane oxygenation cases and mortality. Analysis of the extracorporeal life support organization registry. Am J Respir Crit Care Med.

[68] Bellezzo JM, Shinar Z, Davis DP, Jaski BE, Chillcott S, Stahovich M (2012). Emergency physician-initiated extracorporeal cardiopulmonary resuscitation. Resuscitation.

[69] Zangrillo A, Landoni G, Biondi-Zoccai G, Greco M, Greco T, Frati G (2013). A meta-analysis of complications and mortality of extracorporeal membrane oxygenation. Crit Care Resusc.

[70] Aziz F, Brehm CE, El-Banyosy A, Han DC, Atnip RG, Reed AB (2014). Arterial complications in patients undergoing extracorporeal membrane oxygenation via femoral cannulation. Ann Vascular Surg.

[71] Risnes I, Wagner K, Nome T, Sundet K, Jensen J, Hynas IA (2006). Cerebral outcome in adult patients treated with extracorporeal membrane oxygenation. Ann Thorac Surg.

[72] Mateen FJ, Muralidharan R, Shinohara RT, Parisi JE, Schears GJ, Wijdicks EF (2011). Neurological injury in adults treated with extracorporeal membrane oxygenation. Arch Neurol.

[73] Hodgson CL, Hayes K, Everard T, Nichol A, Davies AR, Bailey MJ (2012). Long-term quality of life in patients with acute respiratory distress syndrome requiring extracorporeal membrane oxygenation for refractory hypoxaemia. Crit Care.

[74] Schmidt M, Zogheib E, Roze H, Repesse X, Lebreton G, Luyt CE (2013). The PRESERVE mortality risk score and analysis of long-term outcomes after extracorporeal membrane oxygenation for severe acute respiratory distress syndrome. Intensive Care Med.

[75] Peek GJ, Elbourne D, Mugford M, Tiruvoipati R, Wilson A, Allen E (2010). Randomised controlled trial and parallel economic evaluation of conventional ventilatory support versus extracorporeal membrane oxygenation for severe adult respiratory failure (CESAR). Health Technol Assess.

